# Hexachlorocyclotriphosphazene (HCCP)-Mediated Direct Formation of Thioethers and Ethers from Quinazolin-4(3*H*)-ones

**DOI:** 10.3390/molecules18055580

**Published:** 2013-05-15

**Authors:** Baoxiang Hu, Xiaochu Zhang, Lili Sheng, Ming Guo, Zhenlu Shen, Xinquan Hu, Nan Sun, Weimin Mo

**Affiliations:** 1 State Key Laboratory Breeding Base of Green Chemistry-Synthesis Technology, College of Chemical Engineering and Materials Science, Zhejiang University of Technology, Hangzhou 310014, China; 2 Department of Chemistry, Zhejiang Agriculture & Forestry University, Lin’An 311300, China

**Keywords:** quinazolin-4(3*H*)-ones, hexachlorocyclotriphosphazene, 4-arylthioquinazolines, 4-aryloxyquinazolines, 4-alkoxyquinazolines

## Abstract

A hexachlorocyclotriphosphazene (HCCP)-mediated direct formation of quinazoline (thio)ethers from quinazolin-4(3*H*)-ones has been developed. Treatment of quinazolin-4(3*H*)-ones with HCCP, diisopropylethylamine (DIPEA), and thiophenols resulted in formation of the corresponding 4-arylthioquinazoline derivatives in moderate to excellent yields. This method has also been utilized to prepare 4-aryloxyquinazoline and 4-alkoxyquinazoline derivatives using phenols and sodium alkoxides as the nucleophiles.

## 1. Introduction

Quinazoline derivatives are an important class of nitrogen-containing heterocycles. They have attracted interest in the past because of their varied biological activities, such as anticonvulsant, antihypertensive, vasodilator, antiinflammatory, antibiosis, fibrinogen receptor antagonistic and nanomolar Hedgehog-antagonistic properties [1,2,3,4,5,6,7,8]. Among the family of quinazolines, quinazoline (thio)ethers, including 4-arylthioquinazolines and 4-aryloxyquinazolines, have received considerable interest because of their potential pharmacological activity [9,10,11,12,13,14,15,16].

Generally, 4-arylthioquinazolines and 4-aryloxyquinazolines are obtained through S_N_Ar substitution of electron-deficient 4-chloroquinazolines with the appropriate thiophenols or phenols in the presence of a base [9,10,11,12,13,14,15,16,17,18,19]. The common method for preparation of 4-chloroquinazolines is the chlorination of corresponding quinazolin-4(3*H*)-ones. The chlorination reagents used include SOCl_2_, POCl_3_, PCl_5_ or their combinations, and the chlorination reactions are usually performed under harsh conditions. However, these chlorination reagents are not environmentally friendly, and some sensitive functional groups may be destroyed under the chlorination conditions. In addition, many 4-chloroquinazoline derivatives are moisture sensitive, thus special treatments are required for their purification and storage.

In order to avoid the use of chlorination reagents and an individual activation step, an *in situ* activation of quinazolin-4(3*H*)-ones is highly desirable. Phosphonium compounds, such as benzotriazol-1-yloxytris(dimethylamino)phosphonium hexafluorophosphate (BOP), have been developed to activate quinazolin-4(3*H*)-ones and successfully employed in the synthesis of 4-aminoquinazolines, 4-arylthioquinazolines and 4-aryloxyquinazolines using amine, thiophenol and phenol nucleophiles, respectively [20,21]. However, BOP and other phosphonium reagents are expensive, moreover, utilization of BOP generates as an end product HMPA, a highly carcinogenic chemical.

**Scheme 1:**
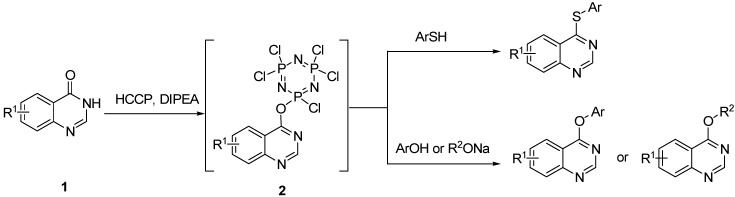
One-pot synthesis of quinazoline (thio)ethers from quinazolin-4(3*H*)-ones mediated by HCCP.

As a part of our research program focused on quinazoline chemistry [22,23], we have recently, reported that hexachlorocyclotriphosphazene (Cl_6_N_3_P_3_, HCCP) can be used as an inexpensive and readily available activating reagent in the direct amination of quinazolin-4(3*H*)-ones (**1**) [24]. In the reaction, quinazolin-4(3*H*)-ones are activated *in situ* with HCCP to generate the highly reactive phosphonium intermediate **2**, then amines can nucleophilically attack **2** to form 4-aminoquinazolines. Thus, we speculated that other nucleophiles, such as thiophenol and phenol ones, might attack **2** to form the corresponding products. Herein, we report a HCCP-mediated, single-pot, facile synthesis of thioethers and ethers from quinazolin-4(3*H*)-ones in moderate to excellent yields (Scheme 1).

## 2. Results and Discussion

Initially, the reaction of quinazolin-4(3*H*)-one (**1a**) and thiophenol was investigated as the model reaction to establish the feasibility of the strategy and to optimize the reaction conditions. The effects of solvent, base, temperature and HCCP loading, *etc*., were evaluated for this model reaction, and the results are summarized in Table 1.

**Table 1:** Optimization of reaction conditions ^a^. 
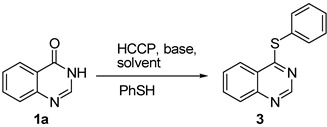

Entry	Base	Solvent	Temperature (°C)	Yield (%) ^b^
1	TEA	MeCN	45	48
2	K_2_CO_3_	MeCN	45	32
3	Cs_2_CO_3_	MeCN	45	69
4	DIPEA	MeCN	45	81
5	DIPEA	THF	45	12
6	DIPEA	DMF	45	23
7	DIPEA	CH_2_Cl_2_	45	7
8	DIPEA	MeCN	45	76 ^c^
9	DIPEA	MeCN	25	68
10	DIPEA	MeCN	65	55
11	DIPEA	MeCN	45	79 ^d^
12	DIPEA	MeCN	45	71 ^e^
13	DIPEA	MeCN	45	81 ^f^
14	DIPEA	MeCN	45	70 ^g^

^a^ Conditions: **1a** (0.5 mmol), HCCP (1.1 equiv.), base (5.0 equiv.), solvent (5 mL), rt, activation time (1 h), then thiophenol (6.0 equiv.), 45 °C, 23 h; ^b^ Isolated yield; ^c^ HCCP (1.0 equiv.); ^d^ Thiophenol (5.0 equiv.); ^e^ Thiophenol (4.0 equiv.); ^f^ DIPEA (6.0 equiv.); ^g^ DIPEA (4.0 equiv.).

Compared with triethylamine (TEA), K_2_CO_3_ and Cs_2_CO_3_ (entries 1–3), diisopropylethylamine (DIPEA) was found to be the most effective in the model reaction of **1a** and thiophenol (entry 4). The results in Table 1 show that the solvent can greatly affect the reaction, and acetonitrile led to much better yield of product **3** than tetrahydrofuran, *N,N*-dimethylformamide or dichloromethane (entries 4–7). According to Scheme 1, 1.0 equiv. HCCP loading was enough, but we found that increasing the HCCP loading from 1.0 to 1.1 equiv. resulted in 5% yield increases (entry 8 *versus* entry 4). After *in situ* activation of **1a** with HCCP at room temperature for 1 h, the S_N_Ar substitution of phosphonium intermediate **2a** (R^1^ = H) with thiophenol was performed at 45 °C. At both lower and higher temperature, the yield of **3** decreased (entries 9 and 10). Like the HCCP-mediated direct amination of quinazolin-4(3*H*)-ones with amines [24], when the thiophenol nucleophile attacks **2a**, two competitive S_N_Ar substitutions were possible, either on the C–O bond or P–Cl bond. Thus, five to six equiv. of thiophenol and DIPEA were needed in this reaction. It was found variation of the amount of thiophenol or DPIEA from 6.0 equiv. to 5.0 equiv. did not significantly change the results (entry 4 *versus* entries 11 and 13), whereas upon decreasing the amount of thiophenol or DPIEA to 4.0 equiv., the yield of **3** was obviously decreased (entries 12 and 14). On the basis of these experimental data, the optimal reaction conditions were: 1.1 equiv. of HCCP, 5.0 equiv. of DIPEA, 5.0 equiv. of thiophenol, 45 °C of reaction temperature and acetonitrile as the reaction solvent, and these were employed in the following studies.

The results of HCCP-mediated direct formation of thioethers (4-arylthioquinazolines) from 1 and thiophenols are summarized in Table 2. Different substituted thiophenols smoothly reacted with **1a** to afford the desired 4-arylthioquinazolines in moderate to excellent yields (entries 1–9). When *o*, *m*, and *p*-methoxythiophenols were used as the nucleophiles, *o*-methoxythiophenol gave the highest product yield, while *m*-methoxythiophenol showed the lowest reactivity (entries 4–6). It is noteworthy that chlorothiophenols, which usually exhibit lower nucleophilicity than methoxythiophenols, could conveniently undergo the transformation and gave excellent product yields in this HCCP-mediated direct formation of 4-arylthioquinazolines (entries 7 and 9). The reactions between thiophenol and a variety of substituted quinazolin-4(3*H*)-ones were also investigated. An array of substituted quinazolin-4(3*H*)-ones were suitable for this HCCP-mediated formation of thioethers and gave the desired 4-arylthioquinazolines in moderate to good yields (entries 1, 10–15). When 5-methylquinazolin-4(3*H*)-one (**1b**) and 5-chloroquinazolin-4(3*H*)-one (**1e**) were used as the substrates, activation time should be prolonged (entries 10 and 13).

**Table 2:** HCCP-mediated formation of quinazoline thioethers from quinazolin-4(3*H*)-ones ^a^. 
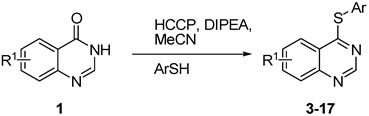

Entry	Quinazolin-4(3*H*)-one		ArSH	Product		Yield (%) ^b^
1	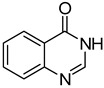	**1a**	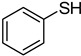	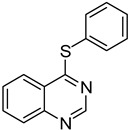	**3**	79
2	**1a**		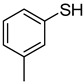	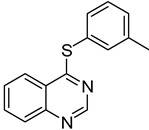	**4**	64 ^c^
3	**1a**		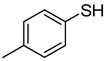	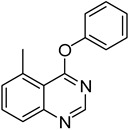	**5**	69
4	**1a**		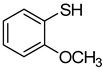	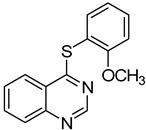	**6**	59
5	**1a**		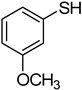	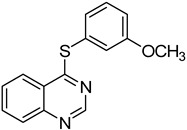	**7**	50
6	**1a**		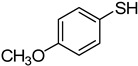	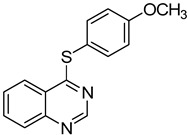	**8**	86
7	**1a**		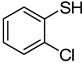	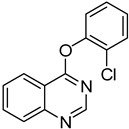	**9**	94
8	**1a**		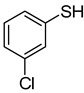	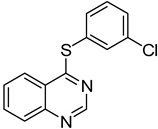	**10**	66
9	**1a**		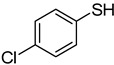	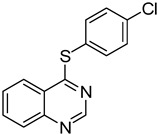	**11**	91
10	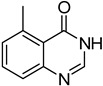	**1b**	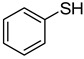	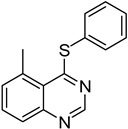	**12**	54 ^d^
11	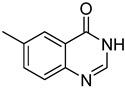	**1c**	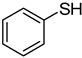	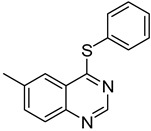	**13**	60
12	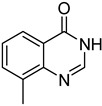	**1d**	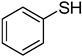	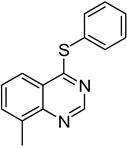	**14**	64
13	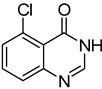	**1e**	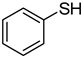	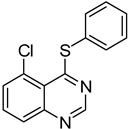	**15**	51 ^d^
14	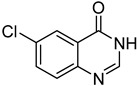	**1f**	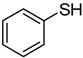	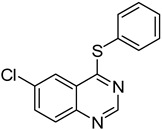	**16**	79
15	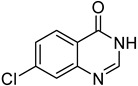	**1g**	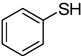	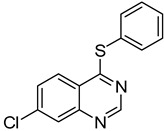	**17**	72

^a^*Reagents andConditions*: **1** (0.5 mmol), HCCP (1.1 equiv.), DIPEA (5.0 equiv.), MeCN (5 mL), rt, activation time (1 h), then thiophenols (5.0 equiv.), 45 °C, 23 h; ^b^ Isolated yield; ^c^*m*-CH_3_PhSH (6.0 equiv.); reaction time (48 h); ^d^ Activation time (20 h).

The method was further extended to the synthesis of 4-aryloxyquinazolines through the reaction of **1** and phenols under the same reaction conditions (Table 3). Different phenols (phenol, *m*-methylphenol, *o*-chlorophenol and *p*-chlorophenol) could react with **1b** and afforded the corresponding products **18**–**21** in moderate to good yields (entry 1–5). There is no significant electronic effect of substituents for products. Substituted quinazolin-4(3*H*)-ones were also used as the substrates to react with phenol. 6-Chloroquinazolin-4(3*H*)-one (**1f**) and 7-chloroquinazolin-4(3*H*)-one (**1g**) gave products **14** and **15** in 70% yield (entries 6 and 7), whereas in the case of 5-methylquinazolin-4(3*H*)-one (**1b**), the product **12** was obtained in 54% yield (entry 5). Alcohols were also used as the nucleophiles in this reaction. Unfortunately, no desired 4-alkoxyquinazolines were formed. It might be due to that the nucleophilicity of alcohols was too weak to undego S_N_Ar substitution. Thus, sodium alkoxides were further utilized as the nucleophiles in this reaction to give the corresponding 4-alkoxyquinazolines (Table 3). When **1a**, 8-methylquinazolin-4(3*H*)-one (**1d**) and **1g** reacted with sodium ethoxide for 3 h, the yields of products 4-ethoxyquinazolines were 54%, 64% and 67%, respectively (entries 8,10 and 11), while **1b** gave only 33% product yield (entry 9). 4-Propoxyquinazoline (**29**) could be obtained in 48% yield in the reaction between **1a** and sodium propoxide (entry 12).

**Table 3:** HCCP-mediated formation of quinazoline ethers from quinazolin-4(3*H*)-ones ^a^. 
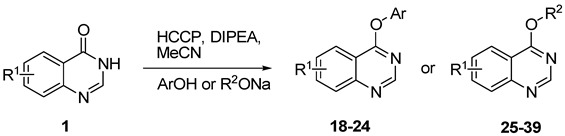

Entry	Quinazolin-4(3*H*)-one	ArOH or RONa	Product		Yield (%) ^b^
1	**1a**	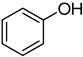	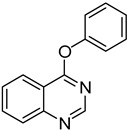	**18**	75
2	**1a**	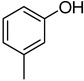	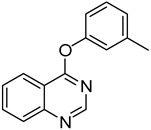	**19**	52
3	**1a**	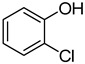	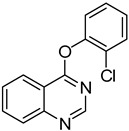	**20**	73
4	**1a**	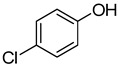	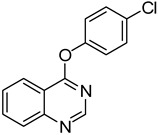	**21**	51
5	**1b**	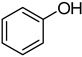	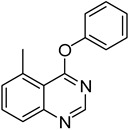	**22**	53 ^c^
6	**1f**	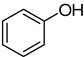	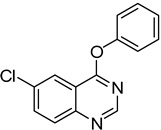	**23**	70
7	**1g**	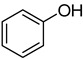	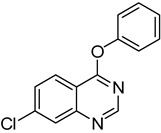	**24**	70
8	**1a**	CH_3_CH_2_ONa	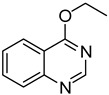	**25**	54
9	**1b**	CH_3_CH_2_ONa	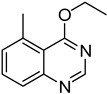	**26**	33 ^c^
10	**1d**	CH_3_CH_2_ONa	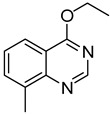	**27**	67
11	**1g**	CH_3_CH_2_ONa	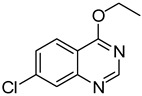	**28**	64
12	**1a**	CH_3_CH_2_CH_2_ONa	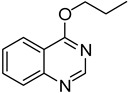	**29**	48

^a^ *Reagents and Conditions*: **1** (0.5 mmol), HCCP (1.1 equiv.), DIPEA (5.0 equiv.), MeCN (5 mL), rt, activation time (1 h), then phenols (5.0 equiv.), 45 °C, 23 h; or R^2^ONa (5.0 equiv.), 45 °C, 3 h; ^b^ Isolated yield; ^c^ Activation time (20 h).

## 3. Experimental

### 3.1. General

^1^H-NMR (500 MHz) and ^13^C-NMR (125 MHz) spectra were obtained on a Bruker Avance III spectrometer. CDCl_3_ and DMSO-*d*_6_ were used as the solvent with tetramethylsilane (TMS) as the internal standard. Low and high resolution mass spectra were recorded in the ESI mode on an Agilent 6210 LC/TOF mass spectrometer. Melting points were measured using XRL-1 melting point instrument and are uncorrected. Quinazolin-4(3*H*)-ones were synthesized from anthranilic acids and formamidine acetate, and their structures were confirmed by MS, ^1^H-NMR, and ^13^C-NMR. Other reagents were purchased from supplier and used without any further treatment.

### 3.2. General Procedure for HCCP-Mediated Formation of Thioethers and Ethers from Quinazolin-4(3H)-ones

Quinazolin-4(3*H*)-ones (**1**, 0.5 mmol), HCCP (171.2 mg, 0.55 mmol, 1.1 equiv.), DIPEA (323.8 mg, 2.5 mmol, 5 equiv.), and CH_3_CN (5 mL) were added to a nitrogen purged vial. The reaction mixture was stirred at room temperature for 1 h as activation time. Then nucleophile (2.5 mmol, 5 equiv. was added, and the reaction mixture was stirred at 45 °C for 23 h. The reaction mixture was partially concentrated under reduced pressure. The crude product was separated on a silica gel plate with ethyl acetate–hexane (1:10 or 1:5) as eluent. Then the area corresponding to the product was scraped off the TLC plate, and extracted with dichloromethane. The extract was concentrated to afford the corresponding products (**3**–**29**).

*4-(Phenylthio)quinazoline* (**3**). White solid (79% yield); mp 109–110 °C; ^1^H-NMR (DMSO-*d6*) δ 8.84 (s, 1H), 8.27 (d, *J* = 8.5 Hz, 1H), 8.07–8.04 (m, 1H), 8.00–7.99 (m, 1H), 7.83–7.79 (m, 1H), 7.68–7.66 (m, 2H), 7.56–7.54 (m, 3H); ^13^C-NMR (DMSO-*d6*) δ 170.3, 153.5, 147.8, 135.8, 134.6, 129.9, 129.6, 128.5, 128.4, 126.5, 123.6, 122.3; HRMS (ESI), *m/z*, 239.0644 [MH^+^], calcd for C_14_H_11_N_2_S, 239.0643.

*4-(m-Tolylthio)quinazoline* (**4**). White solid (64% yield); mp 58–61 °C; ^1^H-NMR (DMSO-*d6*) δ 8.83 (s, 1H), 8.25 (d, *J* = 8.0 Hz, 1H), 8.05–8.02 (m, 1H), 7.99–7.97 (m, 1H), 7.81–7.77 (m, 1H), 7.48–7.41 (m, 3H), 7.36–7.35 (m, 1H), 2.37 (s, 3H); ^13^C-NMR (DMSO-*d6*) δ 170.5, 153.5, 147.8, 139.0, 136.0, 134.6, 132.9, 130.6, 129.4, 128.5, 128.3, 126.2, 123.6, 122.4, 20.7; HRMS (ESI), *m/z*, 253.0795 [MH^+^], calcd for C_15_H_13_N_2_S, 253.0799.

*4-(p-Tolylthio)quinazoline* (**5**) White solid (69% yield); mp 104–105 °C; ^1^H-NMR (DMSO-*d6*) δ 8.81 (s, 1H), 8.19 (d, *J* = 8.0 Hz, 1H), 8.02–7.98 (m, 1H), 7.96–7.95 (m, 1H), 7.77–7.73 (m, 1H), 7.50–7.48 (m, 2H), 7.31 (d, *J* = 8.0 Hz, 2H), 2.38 (s, 3H); ^13^C-NMR (DMSO-*d6*) δ 170.6, 153.4, 147.7, 139.7, 135.7, 134.4, 130.1, 128.4, 128.2, 123.4, 122.9, 122.3, 20.8; HRMS (ESI), *m/z*, 253.0798 [MH^+^], calcd for C_15_H_13_N_2_S, 253.0799. 

*4-(2-Methoxyphenylthio)quinazoline* (**6**). White solid (59% yield); mp 111–113 °C; ^1^H-NMR (DMSO-*d6*) δ 8.80 (s, 1H), 8.27 (d, *J* = 8.0 Hz, 1H), 8.04–8.01 (m, 1H), 7.98–7.96 (m, 1H), 7.80–7.76 (m, 1H), 7.60–7.54 (m, 2H), 7.20 (d, *J* = 8.0 Hz, 1H), 7.10–7.07 (m, 1H), 3.74 (s, 3H); ^13^C-NMR (DMSO-*d6*) δ 170.0, 160.0, 153.5, 147.8, 137.5, 134.4, 132.2, 128.4, 128.2, 123.7, 122.5, 121.2, 114.2, 112.3, 55.9; HRMS (ESI), *m/z*, 269.0747 [MH^+^], calcd for C_15_H_13_N_2_OS, 269.0749.

*4-(3-Methoxyphenylthio)quinazoline* (**7**). White solid (50% yield); mp 80–82 °C; ^1^H-NMR (DMSO-*d6*) δ 8.86 (s, 1H), 8.25 (d, *J* = 8.0 Hz, 1H), 8.06–8.03 (m, 1H), 8.00–7.98 (m, 1H), 7.81–7.78 (m, 1H), 7.47–7.44 (m, 1H), 7.24–7.22 (m, 2H), 7.14–7.11 (m, 1H), 3.80 (s, 3H); ^13^C-NMR (DMSO-*d6*) δ 170.3, 159.7, 153.5, 147.8, 134.6, 130.3, 128.5, 128.3, 127.8, 127.5, 123.5, 122.4, 120.9, 115.7, 55.3; HRMS (ESI), *m/z*, 269.0746 [MH^+^], calcd for C_15_H_13_N_2_OS, 269.0749.

*4-(4-Methoxyphenylthio)quinazoline* (**8**). White solid (86% yield); mp 125–128 °C; ^1^H-NMR (DMSO-*d6*) δ 8.82 (s, 1H), 8.24 (d, *J* = 8.0 Hz, 1H), 8.04–8.01 (m, 1H), 7.98–7.96 (m, 1H), 7.80–7.77 (m, 1H), 7.57–7.54 (m, 2H), 7.10–7.09 (m, 2H), 3.84 (s, 3H); ^1^^3^C-NMR (DMSO-*d6*) δ 171.1, 160.7, 153.5, 147.7, 137.6, 134.6, 128.4, 128.3, 123.6, 122.3, 116.6, 115.3, 55.4; HRMS (ESI), *m/z*, 269.0739 [MH^+^], calcd for C_15_H_13_N_2_OS, 269.0749.

*4-(2-Chlorophenylthio)quinazoline* (**9**). White solid (94% yield); mp 124–126 °C; ^1^H-NMR (DMSO-*d6*) δ 8.85 (s, 1H), 8.29 (d, *J* = 8.0 Hz, 1H), 8.08–8.05 (m, 1H), 8.02–8.00 (m, 1H), 7.84–7.80 (m, 2H), 7.74–7.72 (m, 1H), 7.62–7.59 (m, 1H), 7.52–7.49 (m, 1H); ^1^^3^C-NMR (DMSO-*d6*) δ 168.9, 153.4, 147.9, 138.8, 138.4, 134.7, 132.2, 130.3, 128.51, 128.47, 128.2, 125.9, 123.6, 122.3; HRMS (ESI), *m/z*, 273.0244 [MH^+^], calcd for C_14_H_10_ClN_2_S, 273.0253.

*4-(3-Chlorophenylthio)quinazoline* (**10**). White solid (66% yield); mp 125–126 °C; ^1^H-NMR (DMSO-*d6*) δ 8.86 (s, 1H), 8.23 (d, *J* = 8.0 Hz, 1H), 8.04–8.03 (m, 1H), 8.00–7.98 (m, 1H), 7.81–7.76 (m, 2H), 7.65–7.61 (m, 2H), 7.58–7.55 (m, 1H); ^1^^3^C-NMR (DMSO-*d6*) δ 169.7, 153.4, 147.9, 135.0, 134.7, 134.4, 133.6, 131.1, 129.9, 128.8, 128.5, 128.4, 123.5, 122.3; HRMS (ESI), *m/z*, 273.0243 [MH^+^], calcd for C_14_H_10_ClN_2_S, 273.0253.

*4-(4-Chlorophenylthio)quinazoline* (**11**). White solid (91% yield); mp 133-134 °C; ^1^H-NMR (DMSO-*d6*) δ 8.85 (s, 1H), 8.24 (d, *J* = 8.0 Hz, 1H), 8.07–8.03 (m, 1H), 8.00–7.99 (m, 1H), 7.82–7.79 (m, 1H), 7.70–7.67 (m, 2H), 7.62–7.60 (m, 2H); ^1^^3^C-NMR (DMSO-*d6*) δ 169.9, 153.4, 147.8, 137.5, 135.0, 134.7, 129.6, 128.5, 128.4, 125.5, 123.5, 122.3; HRMS (ESI), *m/z*, 273.0240 [MH^+^], calcd for C_14_H_10_ClN_2_S, 273.0253.

*5-Methyl-4-(phenylthio)quinazoline* (**12**). White solid (54% yield); mp 131–133 °C; ^1^H-NMR (DMSO-*d6*) δ 8.66 (s, 1H), 7.85–7.82 (m, 1H), 7.80–7.78 (m, 1H), 7.62–7.57 (m, 3H), 7.55–7.52 (m, 3H), 3.07 (s, 3H); ^1^^3^C-NMR (DMSO-*d6*) δ 171.3, 152.2, 150.2, 135.9, 135.4, 133.4, 130.8, 129.7, 129.5, 128.2, 127.0, 123.4, 24.7; HRMS (ESI), *m/z*, 253.0790 [MH^+^], calcd for C_15_H_13_N_2_S, 253.0799.

*6-Methyl-4-(phenylthio)quinazoline* (**13**). White solid (60% yield); mp 100–102 °C; ^1^H-NMR (DMSO-*d6*) δ 8.83 (s, 1H), 8.00 (s, 1H), 7.89 (d, *J* = 8.5 Hz, 1H), 7.73 (d, *J* = 8.5 Hz, 1H), 7.67–7.65 (m, 2H), 7.52–7.51 (m, 3H), 2.62 (s, 3H); ^1^^3^C-NMR (DMSO-*d6*) δ 170.4, 153.3, 146.9, 137.9, 136.0, 135.8, 129.7, 129.4, 128.6, 127.4, 123.3, 122.7, 21.8; HRMS (ESI), *m/z*, 253.0796 [MH^+^], calcd for C_15_H_13_N_2_S, 253.0799.

*8-Methyl-4-(phenylthio)quinazoline* (**14**). White solid (64% yield); mp 98–100 °C; ^1^H-NMR (DMSO-*d6*) δ 8.84 (s, 1H), 8.04 (d, *J* = 8.0 Hz, 1H), 7.85 (d, *J* = 7.0 Hz, 1H), 7.65–7.62 (m, 3H), 7.54-7.53 (m, 3H), 2.66 (s, 3H); ^1^^3^C-NMR (DMSO-*d6*) δ 170.3, 152.6, 146.8, 136.7, 135.7, 134.2, 129.7, 129.5, 127.7, 126.8, 122.2, 121.1, 17.0; HRMS (ESI), *m/z*, 253.0789 [MH^+^], calcd for C_15_H_13_N_2_S, 253.0799.

*5-Chloro-4-(phenylthio)quinazoline* (**15**). White solid (51% yield); mp 133–134 °C; ^1^H-NMR (CDCl_3_) δ 8.73 (s, 1H), 7.91–7.89 (m, 1H), 7.74–7.68 (m, 2H), 7.60–7.58 (m, 2H), 7.51–7.50 (m, 3H); ^1^^3^C-NMR (CDCl_3_) δ 172.5, 153.2, 151.3, 135.9, 132.7, 130.6, 129.9, 129.7, 129.4, 129.3, 128.5, 122.6; HRMS (ESI), *m/z*, 273.0244 [MH^+^], calcd for C_14_H_10_ClN_2_S, 273.0253.

*6-Chloro-4-(phenylthio)quinazoline* (**16**). White solid (79% yield); mp 103–105 °C; ^1^H-NMR (CDCl_3_) δ 8.86 (s, 1H), 8.22 (d, *J* = 2.0 Hz, 1H), 7.93 (d, *J* = 9.0 Hz, 1H), 7.83-7.81 (m, 1H), 7.66–7.64 (m, 2H), 7.53–7.51 (m, 3H); ^1^^3^C-NMR (CDCl_3_) δ 170.7, 154.0, 146.9, 135.8, 134.8, 133.3, 130.5, 130.0, 129.5, 126.7, 123.8, 122.9; HRMS (ESI), *m/z*, 273.0241 [MH^+^], calcd for C_14_H_10_ClN_2_S, 273.0253.

*7-Chloro-4-(phenylthio)quinazoline* (**17**). White solid (72% yield); mp 142–143 °C; ^1^H-NMR (DMSO-*d6*) δ 8.85 (s, 1H), 8.29 (d, *J* = 9.0 Hz, 1H), 8.07 (d, *J* = 2.0 Hz, 1H), 7.83–7.81 (m, 1H), 7.67–7.66 (m, 2H), 7.57–7.55 (m, 3H); ^1^^3^C-NMR (DMSO-*d6*) δ 170.7, 154.5, 148.6, 139.2, 135.7, 130.0, 129.6, 128.9, 127.3, 126.1, 125.7, 121.0; HRMS (ESI), *m/z*, 273.0255 [MH^+^], calcd for C_14_H_10_ClN_2_S, 273.0253. 

*4-Phenoxyquinazoline* (**18**). White solid (75% yield); mp 123–125 °C; ^1^H-NMR (CDCl_3_) δ 8.80 (s, 1H), 8.42–8.40 (m, 1H), 8.03 (d, *J* = 8.5 Hz, 1H), 7.96–7.94 (m, 1H), 7.69 (t, *J* =1.0 Hz, 1H), 7.54–7.50 (m, 2H), 7.37–7.35 (m, 1H), 7.30–7.28 (m, 2H); ^1^^3^C-NMR (CDCl_3_) δ 167.0, 154.4, 152.4, 151.7, 134.2, 129.8, 128.0, 127.6, 126.1, 123.7, 121.9, 116.5; HRMS (ESI), *m/z*, 223.0868 [MH^+^], calcd for C_14_H_11_N_2_O, 223.0871.

*4-(m-Tolyloxy)quinazoline* (**19**). White solid (52% yield); mp 100–101 °C; ^1^H-NMR (CDCl_3_) δ 8.81 (s, 1H), 8.41–8.39 (m, 1H), 8.03 (d, *J* = 8.5 Hz, 1H), 7.95–7.92 (m, 1H), 7.70–7.67 (m, 1H), 7.41–7.38 (d, *J* = 7.5 Hz, 1H), 7.17–7.08 (m, 3H), 2.44 (s, 3H); ^1^^3^C-NMR (CDCl_3_) δ 167.1, 154.4, 152.3, 151.6, 140.1, 134.1, 129.5, 127.9, 127.6, 126.9, 123.7, 122.4, 118.8, 116.5, 21.4; HRMS (ESI), *m/z*, 237.1025 [MH^+^], calcd for C_15_H_13_N_2_O, 237.1028.

*4-(2-Chlorophenoxy)quinazoline* (**20**). White solid (73% yield); mp 118–119 °C; ^1^H-NMR (CDCl_3_) δ 8.78 (s, 1H), 8.46–8.45 (m, 1H), 8.05 (d, *J* = 8.5 Hz, 1H), 7.98–7.96 (m, 1H), 7.74–7.72 (m, 1H), 7.57–7.56 (m, 1H), 7.43–7.41 (m, 1H), 7.38–7.36 (m, 1H), 7.34–7.32 (m, 1H); ^1^^3^C-NMR (CDCl_3_) δ 166.2, 154.1, 151.9, 148.6, 134.3, 130.7, 128.1, 128.0, 127.8, 127.31, 127.26, 124.1, 123.7, 116.1; HRMS (ESI), *m/z*, 257.0479 [MH^+^], calcd for C_14_H_10_ClN_2_O, 257.0482.

*4-(4-Chlorophenoxy)quinazoline* (**21**). White solid (51% yield); mp 105–108 °C; ^1^H-NMR (CDCl_3_) δ 8.79 (s, H), 8.39 (d, *J* = 8.0 Hz, 1H), 8.04 (d, *J* = 8.5 Hz, 1H), 7.97–7.94 (m, 1H), 7.72–7.69 (m, 1H), 7.49–7.46 (m, 2H), 7.28–7.23 (m, 2H); ^1^^3^C-NMR (CDCl_3_) δ 116.8, 154.1, 151.8, 150.9, 134.3, 131.5, 129.9, 128.0, 127.8, 123.5, 123.3, 116.3; HRMS (ESI), *m/z*, 257.0469 [MH^+^], calcd for C_14_H_10_ClN_2_O, 257.0482. 

*5-Methyl-4-phenoxyquinazoline* (**22**). White solid (53% yield); mp 73–75 °C; ^1^H-NMR (CDCl_3_) δ 8.70 (s, 1H), 7.86 (d, *J* = 8.5 Hz, 1H), 7.77 (t, *J* = 7.5 Hz, 1H), 7.53–7.50 (m, 2H), 7.45 (d, *J* = 7.5 Hz, 1H), 7.36–7.33 (m, 1H), 7.28–7.25 (m, 2H), 3.00 (s, 3H); ^1^^3^C-NMR (CDCl_3_) δ 168.0, 153.7, 153.4, 152.3, 136.7, 133.4, 129.9, 129.8, 126.1, 125.9, 122.0, 116.3, 24.1; HRMS (ESI), *m/z*, 237.1021 [MH^+^], calcd for C_15_H_13_N_2_O, 237.1028.

*6-Chloro-4-phenoxyquinazoline* (**23**). White solid (70% yield); mp 94–97 °C; ^1^H-NMR (CDCl_3_) δ 8.77 (s, 1H), 8.37 (d, *J* = 2.0 Hz, 1H), 7.96 (d, *J* = 9.0 Hz, 1H), 7.86–7.83 (m, 1H), 7.52–7.49 (m, 2H), 7.36–7.33 (m, 1H), 7.29–7.27 (m, 2H); ^1^^3^C-NMR (CDCl_3_) δ 166.1, 154.4, 152.1, 150.1, 134.9, 133.2, 129.8, 129.6, 126.2, 122.7, 121.7, 117.1; HRMS (ESI), *m/z*, 257.0471 [MH^+^], calcd for C_14_H_10_ClN_2_O, 257.0482.

*7-Chloro-4-phenoxyquinazoline* (**24**). White solid (70% yield); mp 190–192 °C; ^1^H-NMR (CDCl_3_) δ 8.78 (s, 1H), 8.35 (d, *J* = 8.5 Hz, 1H), 8.03 (d, *J* = 2.0 Hz, 1H), 7.65–7.63 (m, 1H), 7.53–7.50 (m, 2H), 7.36 (t, *J* = 7.5 Hz, 1H), 7.29–7.27 ppm (m, 2H); ^1^^3^C-NMR (CDCl_3_) δ 166.9, 155.5, 152.4, 152.2, 140.5, 129.8, 128.7, 127.2, 126.2, 125.2, 121.8, 114.9; HRMS (ESI), *m/z*, 257.0471 [MH^+^], calcd for C_14_H_10_ClN_2_O, 257.0482.

*4-Ethoxyquinazoline* (**25**). White solid (54% yield); mp 31–33 °C; ^1^H-NMR (CDCl_3_) δ 8.80 (s, 1H), 8.19–8.18 (m, 1H), 7.93 (d, *J* = 8.0 Hz, 1H), 7.84–7.81 (m, 1H), 7.58–7.54 (m, 1H), 4.64 (q, *J* = 7.0 Hz, 2H), 1.53 (t, *J* = 7.0 Hz, 3H); ^1^^3^C-NMR (CDCl_3_) δ 166.7, 154.5, 150.9, 133.4, 127.7, 126.9, 123.6, 116.8, 63.1, 14.4; HRMS (ESI), *m/z*, 175.0873 [MH^+^], calcd for C_10_H_11_N_2_O, 175.0871.

*4-Ethoxy-5-methylquinazoline* (**26**). White solid (33% yield); mp 66–69 °C; ^1^H-NMR (CDCl_3_) δ 8.72 (s, 1H), 7.77 (d, *J* = 8.5 Hz, 1H), 7.67 (t, *J* = 7.5 Hz, 1H), 7.33 (d, *J* = 7.5 Hz, 1H), 4.61 (q, *J* = 7.0 Hz, 2H), 2.86 (s, 3H), 1.54 (t, *J* = 7.0 Hz, 3H); ^1^^3^C-NMR (CDCl_3_) δ 167.8, 153.8, 152.6, 136.9, 132.7, 129.3, 125.8, 116.4, 63.1, 24.0, 14.4; HRMS (ESI), *m/z*, 189.1034 [MH^+^], calcd for C_11_H_13_N_2_O, 189.1028.

*4-Ethoxy-8-methylquinazoline* (**27**). White solid (67% yield); mp 31–32 °C; ^1^H-NMR (CDCl_3_) δ 8.84 (s, 1H), 8.04–8.02 (m, 1H), 7.66 (d, *J* = 7.5 Hz, 1H), 7.44 (t, *J* = 8.0 Hz, 1H), 4.63 (q, *J* = 7.0 Hz, 2H), 2.73 (s, 3H), 1.52 (t, *J* = 7.0 Hz, 3H); ^1^^3^C-NMR (CDCl_3_) δ 166.9, 153.4, 150.0, 135.9, 133.5, 126.4, 121.2, 116.6, 62.9, 17.6, 14.3; HRMS (ESI), *m/z*, 189.1023 [MH^+^], calcd for C_11_H_13_N_2_O, 189.1028.

*7-Chloro-4-ethoxyquinazoline* (**28**). White solid (64% yield); mp 87–88 °C; ^1^H-NMR (CDCl_3_) δ 8.76 (s, 1H), 8.08 (d, *J* = 9.0 Hz, 1H), 7.90 (d, *J* = 1.5 Hz, 1H), 7.49–7.47 (m, 1H), 4.62 (q, *J* = 7.0 Hz, 2H), 1.51 (t, *J* = 7.0 Hz, 3H); ^1^^3^C-NMR (CDCl_3_) δ 166.6, 155.5, 151.6, 139.6, 127.8, 126.9, 125.0, 115.0, 63.3, 14.3; HRMS (ESI), *m/z*, 209.0479 [MH^+^], calcd for C_10_H_10_ClN_2_O, 209.0482.

*4-Propoxyquinazoline* (**29**). White solid (48% yield); mp 190–192 °C; ^1^H-NMR (CDCl_3_) δ 8.79 (s, 1H), 8.19–8.17 (m, 1H), 7.92 (d, *J* = 8.5 Hz, 1H), 7.83–7.80 (m, 1H), 7.57–7.53 (m, 1H), 4.52 (t, *J* = 6.5 Hz, 2H), 1.95–1.91 (m, 2H), 1.10 (t, *J* = 7.5 Hz, 3H); ^1^^3^C-NMR (CDCl_3_) δ 166.8, 154.4, 150.9, 133.4, 127.6, 126.9, 123.5, 116.7, 68.7, 22.1, 10.5; HRMS (ESI), *m/z*, 189.1023 [MH^+^], calcd for C_11_H_13_N_2_O, 189.1028.

## 4. Conclusions

In summary, we have successfully developed an HCCP-mediated direct formation of thioethers (4-arylthioquinazolines) from quinazolin-4(3*H*)-ones and thiophenols in moderate to excellent yields. This method has also been utilized to prepare quinazoline ethers, including 4-aryloxyquinazolines and 4-alkoxyquinazolines, using phenols and sodium alkoxides as the nucleophiles. This direct formation of quinazoline (thio)ethers is mild, convenient, and suitable for a wide range of less expensive nucleophiles. This methodology would facilitate the syntheses of 4-arylthioquinazoline and 4-aryloxyquinazoline derivatives in medicinal chemistry.
